# Selective Evolution of Ligands by Exponential Enrichment to Identify RNA Aptamers against Shiga Toxins

**DOI:** 10.1155/2014/214929

**Published:** 2014-04-15

**Authors:** Sreerupa Challa, Saul Tzipori, Abhineet Sheoran

**Affiliations:** ^1^Department of Infectious Disease and Global Health, Tufts Cummings School of Veterinary Medicine, Tufts University, 200 Westboro Road, Building 20, North Grafton, MA 01536, USA; ^2^AstraZeneca, 35 Gatehouse Drive, Waltham, MA 02451, USA

## Abstract

Infection with Shiga toxin- (Stx-) producing *E. coli* causes life threatening hemolytic uremic syndrome (HUS), a leading cause of acute renal failure in children. Of the two antigenically distinct toxins, Stx1 and Stx2, Stx2 is more firmly linked with the development of HUS. In the present study, selective evolution of ligands by exponential enrichment (SELEX) was used in an attempt to identify RNA aptamers against Stx1 and Stx2. After 5 rounds of selection, significant enrichment of aptamer pool was obtained against Stx2, but not against Stx1, using a RNA aptamer library containing 56 random nucleotides (N56). Characterization of individual aptamer sequences revealed that six unique RNA aptamers (mA/pC, mB/pA, mC, mD, pB, and pD) recognized Stx2 in a filter binding assay. None of these aptamers bound Stx1. Aptamers mA/pC, mB/pA, mC, and mD, but not pB and pD, partially blocked binding of Alexa 488-labeled Stx2 with HeLa cells in a flow cytometry assay. However, none of the aptamers neutralized Stx2-mediated cytotoxicity and death of HeLa cells.

## 1. Introduction

Infection with Shiga toxin- (Stx-) producing* Escherichia coli* (STEC) is the most significant cause of hemolytic uremic syndrome (HUS), the leading cause of acute renal failure in children [1–4]. Of the two antigenically distinct toxins, Stx1 and Stx2, Stx2 is more firmly linked with the development of HUS as STEC strains producing this toxin are more frequently associated with HUS than strains that produce both Stx1 and Stx2, while Stx1 alone has rarely been associated with HUS [5–7]. Stx1 and Stx2 are similar in basic structure [8], binding specificity [8], and mode of action. Both toxins consist of an A-subunit monomer and a B-subunit pentamer [8–10]. The pentameric B subunit binds to its cell surface receptor CD77, also called globotriaosylceramide (Gb3; Gal*α*1-4Gal*β*1-4glucosyl ceramide) [11, 12]. This triggers endocytosis of the holotoxin, mainly through clathrin-coated pits [13]. The internalization of catalytically active A-subunit, delivered to cytosol via retrograde transport, causes shut down of protein synthesis and leads to cell death [14, 15].

Nucleic acid aptamers are being developed as therapeutic and diagnostic reagents against biotoxins [16]. Aptamers are short, synthetic, single stranded nucleic acids with unique three-dimensional conformations that bind strongly and selectively to a target molecule via shape specific recognition [17–20]. Aptamers are similar in many respects to antibodies except that they are cheaper to produce, have extremely long shelf lives, and thus, are far more cost effective than to produce antibodies. A single aptamer against a Shiga toxin has been reported, but it is unknown whether the aptamer is against Stx1 or Stx2 [21]. Toxin-neutralizing ability of this toxin is also unknown [21]. The aim of the present study was to utilize selective evolution of ligands by exponential enrichment to identify ribonucleic acid (RNA) aptamers against Stx1 and Stx2.

## 2. Materials and Methods

### 2.1. Stx1 and Stx2

Stx1 and Stx2, purified as described previously [22], were obtained from Phoenix Laboratory (Tufts University, NEMC Microbial Products & Services Facility). Before use, the toxins were passed through Polyacrylamide 6000 Desalting column (Thermoscientific, IL) to suspend them in selection buffer (20 mM HEPES (pH 7.3), 150 mM NaCl, and 5 mM MgCl2). The toxins were quantified by UV spectrophotometry (ND-1000 Spectrophotometer, Nanodrop), aliquoted, and stored at −20°C.

### 2.2. Preparation of RNA Library

The starting ssDNA library containing 56 random nucleotides flanked by constant regions (5′-CACAGCGGGACAGTTTAGC - N56 - GGTAGGTGGGTGCGTCTAAA-3′) was synthesized by Integrated DNA Technologies, Inc. (Coralville, IA). The DNA random library (4 nmoles or approximately 2.4 × 10^15^ sequences in 50 mL, that is, 80 nM) sequences were amplified by PCR with 200 *μ*M of each dNTP along with 2 *μ*M each of forward (5′-GATAATACGACTCACTATAGGGCACAGCGGGACAGTTTAGC-3′) and reverse (5′-TTTAGACGCACCCACCTACC-3′) primers using Taq DNA polymerase (New England Biolabs); the forward primer included a T7 promoter region (underlined) for in vitro transcription. In order to prevent the abundance of the original DNA library, PCR was performed for a total volume of 50 mL (100 *μ*L/tube) and was limited to 10 cycles. After the PCR reactions, the amplified dsDNA library was subjected to ethanol precipitation and later converted to RNA library using AmpliScribe T7-Flash Transcription Kit (Epicentre, Madison, WI) according to the manufacturer's instructions. The in vitro transcription reaction was set up with 12 *μ*L of 65 *μ*M of dsDNA (i.e., 0.8 nmoles or approximately 5 × 10^14^ sequences). The RNA was purified after completion of the transcription reaction and resuspended in 200 *μ*L of dH_2_O. The final concentration of purified RNA was found to be 32 nmoles that would have corresponded to 40 copies of original DNA pool, that is, 5 × 10^14^ sequences (0.8 nmoles).

As unmodified RNA molecules are highly susceptible to nuclease degradation, nuclease resistant modifications were introduced chemically to generate modified RNA aptamer library. Modified library was generated by incorporating 2′-Fluorine-CTP (2′-F-dCTP), 2′-Fluorine-UTP (2′-F-dUTP), ATP, and GTP into full length transcripts with in vitro transcription using DuraScribe T7 transcription kit (Epicentre, Madison, WI, USA) as described elsewhere using Y639F mutant of T7 RNA polymerase [23, 24].

### 2.3. In Vitro Selection of Aptamers Using Nitrocellulose Membrane

Systematic evolution of ligands by exponential enrichment (SELEX) using nitrocellulose membrane (m-SELEX) was performed as described elsewhere [17] to select aptamers against Stx1 and Stx2. In every round, prior to selection, RNA was thermally equilibrated and refolded in selection buffer by heating to 70°C for 3 min and then slowly cooling to 37°C for at least 10 minutes. In order to minimize nonspecific binding of RNA with the nitrocellulose membrane, the refolded RNA pool was preadsorbed to a 0.45 *μ*m nitrocellulose HAWP filter (Millipore, Bedford, MA) for 30 min prior to every round of selection with Stx1 or Stx2.

In the first selection cycle, the precleared random RNA pool (55.2 *μ*g, 1.6 nmole, 9.6 × 10^14^ molecules) and Stx1 or Stx2 (0.4 nmole, RNA/toxin ratio 4/1) were incubated in 200 *μ*L selection buffer on a rotating platform for 30 min at room temperature (RT). From round 2, competitor yeast tRNA (0.375 nmole) was also added to the reaction mixture. In rounds 3 and 4, SELEX was performed with equimolar ratio of RNA and toxin to increase the chances of RNA binding to the toxin. Later to improve the stringency of selection, RNA/toxin ratio was increased to 4/1 and 8/1 in rounds 5 and 7, respectively (Table 1). The toxin-RNA mixture was then passed through a prewetted nitrocellulose filter followed by 5 times of washing with 200 *μ*L of selection buffer. The Stx1/Stx2 bound RNA was eluted from the filter with 400 *μ*L TE Buffer (10 mM TRIS, 1 mM EDTA; pH 8.0) at 95°C for 5 min, followed by ethanol precipitation.

The recovered RNA pool was reverse-transcribed into cDNA in a total volume of 20 *μ*L using MuLV Reverse Transcriptase (Applied Biosystems, CA) and amplified for 12 cycles of PCR followed by ethanol precipitation. Finally, the dsDNA was transcribed in vitro using T7 RNA polymerase and purified on 8% polyacrylamide/7 M urea gels as described above to synthesize the enriched RNA pool to be used for the next round of selection.

### 2.4. In Vitro Selection of Aptamers Using Microtiter Plate

SELEX was also performed using microtiter plate (p-SELEX) to select aptamers against Stx1 and Stx2. The 96-well plate (Evergreen Scientific, Los Angeles, CA) was coated with 20 *μ*g/mL of Stx1-specific mouse monoclonal antibody (MAb) 4D3 or Stx2-specific mouse MAb 3D1 overnight followed by blocking with 1% BSA (Amgen, Thousand Oaks, CA) and binding with 20 *μ*g/mL of Stx1 or Stx2 (28 pmoles), respectively, in the selection buffer (20 mM HEPES (pH 7.4), 150 mM NaCl, 5 mM MgCl2, and 0.1% Tween 20) for 1 h at RT each. BSA was used instead of tRNA to reduce nonspecific binding of both toxins and RNA as described elsewhere for plate selection [25, 26]. Prior to every round of selection, RNA was thermally equilibrated and refolded as described above and preabsorbed with 4D3/BSA- or 3D1/BSA-bound plate to minimize nonspecific binding of RNA to the plate. After preclearing, RNA was incubated with Stx1 or Stx2 in 100 *μ*L of the selection buffer for 1 h at RT followed by washing 6 times with 200 *μ*L tween-selection buffer. Elution of RNA was performed by heating the plate at 95°C for 5 min and reverse transcription was directly done on the plate at 42°C in a total volume of 50 *μ*L with MuLV Reverse Transcriptase (Applied Biosystems) followed by amplification into DNA by PCR with Taq Polymerase (New England Biolabs). Finally, the DNA was transcribed in vitro using T7 RNA polymerase (Epicentre) and purified on 8% polyacrylamide/7 M urea gels to synthesize the enriched RNA pool to be used for the next round of selection.

### 2.5. Nitrocellulose Filter Binding Assay

Nitrocellulose filter binding assay was used to evaluate the RNA pool binding capability after every other round [27]. RNA pools after 0, 1, 3, 5, and 7 rounds of selection were radiolabelled during transcription by incorporation of [*α*-32P]-ATP and subsequently purified on 8% polyacrylamide/7 M urea gels. Thermally equilibrated 10 nM radiolabelled RNA pools were incubated with 0.5 *μ*M of Stx1 or Stx2 or BSA in selection buffer in the presence of 0.1 *μ*g/*μ*L yeast tRNA. The Stx1/Stx2-RNA mixtures were then passed through a vacuum manifold containing nitrocellulose and nylon membranes (the nitrocellulose membrane captures RNA+ protein complexes, while the nylon membrane captures all free RNA that flows through the nitrocellulose membrane) and washed three times with selection buffer. Both nylon and nitrocellulose membranes were exposed to a phosphor screen for at least 4 h and the radioactivity was measured using phosphorimager (Molecular Dynamics Storm Phosphoimager 840, GE/Amersham). The fraction of RNA bound to the Stx1 or Stx2 was calculated with the following formula: fraction bound = cpm on nitrocellulose/(cpm on nitrocellulose + cpm on nylon) × 100.

### 2.6. Cloning and Sequencing

After 7 rounds of selection with no further enrichment observed, the dsDNA from round 5 of SELEX was amplified by PCR and cloned into pCR 2.1-TOPO Vector cloning vector (Invitrogen, Grand Island, NY) and transformed into* E. coli* (Top 10 cells, Invitrogen). Fifty-one individual clones from membrane-SELEX (m-SELEX) and 45 clones from plate-SELEX (p-SELEX) were picked and sequenced using M13 forward primer. According to the alignments of individual sequences, membrane-SELEX aptamers were grouped into 5 groups and ELISA-SELEX aptamers were grouped into 4 groups. One representative sequence from each group was chosen for further analysis because of relative abundance within their group.

### 2.7. HeLa Cell Cytotoxicity Assay

An in vitro HeLa cell cytotoxicity, optimized to evaluate ability of RNA aptamers to neutralize cytotoxic effects of Stx2, was used. HeLa cells were cultured on 96-well plates at 1 × 10^4^ cells/well in DMEM medium with 10% fetal bovine serum overnight at 37°C (complete DMEM medium). After reaching 60–70% cell confluence, cells were washed 4 times with nuclease free phosphate-buffered saline (NF-PBS) (Ambion, TX, USA) before adding Stx2. Stx2 at 0.5 ng/well (0.007 pmole) or 4 ng/well (0.056 pmole) was preincubated either with individual aptamers at 50 *μ*g/well (1550 pmole) or with Stx2-neutralizing HuMAb 5H8 at 1.0 *μ*g/well (6.66 pmole) at 37°C for 30 min before adding to the cells. All reagents were diluted in NF-PBS. Cells were then incubated with either Stx2 alone or Stx2 with RNA aptamers or Stx2 with 5H8 HuMAb at 37°C for 30 min. The supernatants in the wells were collected and run on PAGE to determine degradation of RNA aptamers. The cells were again washed with NF-PBS 4 times and incubated in complete DMEM medium for 24 h at 37°C. The percentage of cell survival in the presence or absence of aptamers and HuMAbs was assessed by crystal violet assay as described [28]. Briefly, cells were washed with PBS and fixed with 4% paraformaldehyde (PF). Crystal violet solution was added to the cells, and after intensive washing with water and drying, the cells were lysed with 100% ethanol. The optical density at 690 nm was measured in a microplate reader. The absorbance (OD) of each well was read at 590 nm. The percentage of cell survival, which is a measurement of the toxin neutralization, was calculated by the following formula: [(OD_toxin  in  presence  of  5H8  or  aptamer_ − OD_toxin  only_)/(OD_no  toxin_ − OD_toxin  only_)] × 100.

### 2.8. Flow Cytometry Analysis

To determine whether Stx2-specific aptamers inhibit binding of Stx2 to its receptor Gb_3_ on the cell surface, flow cytometry analysis was performed as described [29]. HeLa cell suspensions were produced by treating the cells with 0.05% trypsin-53 mM EDTA. Cells were then washed three times with cold NF-PBS by centrifugation at 300 ×g at 4°C for 10 min. AF488-labeled Stx2 (Stx2-AF) at 0.6 *μ*g/mL (100 *μ*L, 0.8 pmole)NF-PBS was preincubated at 37°C for 1 h with aptamers (500 *μ*g/mL NF-PBS, 100 *μ*L, 1550 pmole), cooled to 4°C, and then added to suspensions of 5 × 10^5^ cells which were also precooled to 4°C. After a 30 min incubation at 4°C, the cells were washed twice in cold NF-PBS and resuspended in 4% PF for 15 min at 4°C. The cells were washed and resuspended in PBS. The cell-associated fluorescence (20,000 cells per treatment) was collected by using BD Accuri C6 flow cytometer (BD Biosciences). Histogram analysis was performed by using BD Accuri C6 software.

## 3. Results

### 3.1. Selection of RNA Aptamers for Stx1 and Stx2

**Figure 1:**
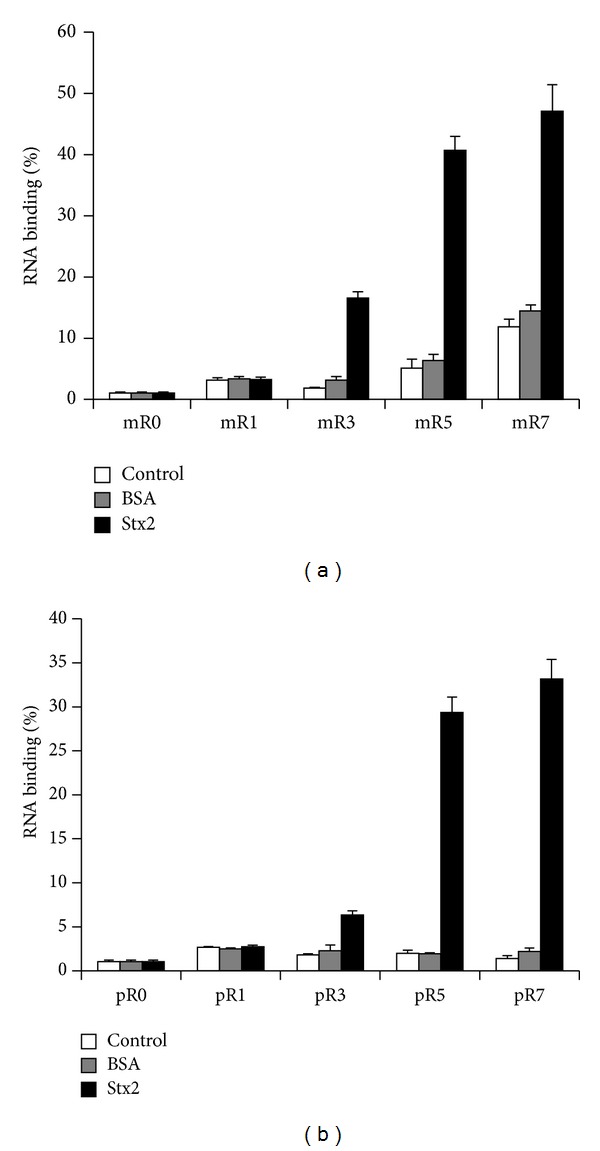
Enrichment of unmodified RNA aptamer pool of 56 random nucleotides for binding with Stx2 through successive selection rounds of SELEX. Binding activity of the RNA pool was analyzed by filter binding assay. Strong enrichment of aptamer pool for Stx2-binders occurred at 5th round of selection by both m-SELEX (a) and p-SELEX (b) as 41% and 29% of the input RNA bound with Stx2, respectively, whereas binding to the membrane and unrelated protein BSA was <5%. The enrichment did not increase with additional rounds of selections. This experiment was repeated twice with similar results.

The filter binding assay was used to monitor the progress of selection after each SELEX cycle. Strong enrichment of N56 (56 random nucleotides) unmodified RNA aptamer pool for Stx2-binders occurred at 5th round of selection by m-SELEX and p-SELEX as 41% and 29% of the input RNA bound with Stx2, respectively, whereas binding to the membrane and unrelated protein BSA was <5% (Figure 1). The enrichment did not increase with additional rounds of selection by m-SELEX as 47% of the input RNA bound with Stx2 in 7th round of selection, but nonspecific RNA binding to the membrane and BSA increased to >12% (Figure 1(a)). Although 7th round of selection by p-SELEX increased enrichment slightly to 33% with no increase in background binding (Figure 1(b)), the enrichment did not increase following additional rounds of selection by p-SELEX (data not shown). Therefore, highly enriched unmodified RNA aptamer pools of 5th selection rounds by m-SELEX and p-SELEX were cloned and sequenced.

Although identical selection conditions were used to perform SELEX as mentioned above with two modified (nuclease resistant) libraries of 35 and 101 random nucleotides, we could not enrich libraries with Stx2-binders (data not shown). Similarly, none of the unmodified or modified libraries showed any enrichment for Stx1-binders even after 12–20 rounds of selection with each library (data not shown).

### 3.2. Analysis of the Selected Stx2 Aptamers

**Figure 2:**
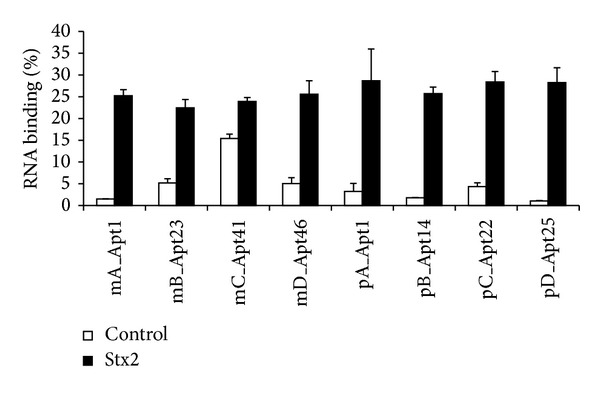
Binding of individual aptamers with Stx2. The binding activity of the individual aptamers with Stx2 was analyzed by filter binding assay. All aptamer sequences showed strong binding with Stx2 except mC_Apt41 which showed moderate binding as it also bound nonspecifically to the membrane more than the other aptamers. This experiment was repeated twice with similar results.

Highly enriched unmodified RNA aptamer pools for Stx2-binders were cloned, and ~100 clones from each pool were sequenced. The individual clones were classified into 5 groups from m-SELEX and 5 groups from p-SELEX based on the alignments of individual aptamer sequences. One representative sequence from each group was chosen for further characterization because of their relative abundance within their group (Table 2). The sequences within each group were almost identical except groups m-singles and p-singles which contained sequences that occurred only once. Seventy-eight percent of m-SELEX sequences were grouped into groups mA and mB and 36% of p-SELEX sequences into groups pA and pC. These groups contained an identical 19-nucleotide (ATTCGATCAGGCAGTACGT) region (Table 2). Furthermore, sequences of groups mA and mB were identical to sequences of groups pC and pA, respectively. However, the other 4 groups (mC, mD, pB, and pD) did not have any similarities between them. Filter binding assay was performed to confirm the binding activity of these selected individual aptamers with Stx2 (Figure 2). Except for the group mC (mC_Apt41), all other groups showed strong specific binding with Stx2. Nonetheless, mC_Apt41 also bound Stx2. None of the aptamers from any group bound Stx1 (results not shown).

#### 3.2.1. Flow Cytometry Analysis

**Figure 3:**
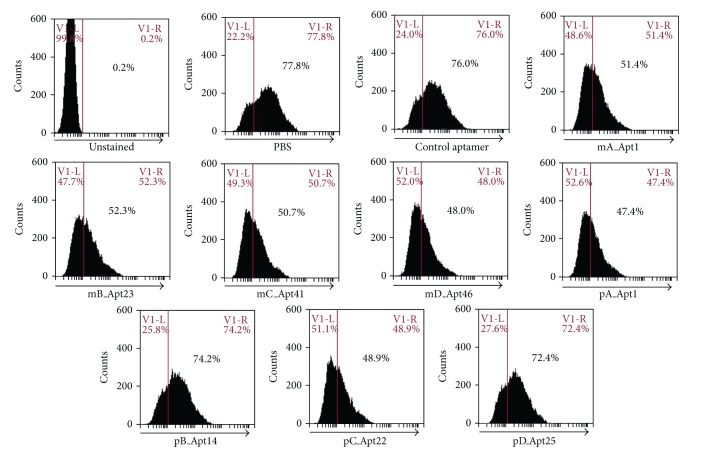
Flow cytometry analysis of HeLa cells treated with Stx2 and aptamers. Flow cytometry analysis showed that, in absence of any aptamer, Stx2-AF labeled ~78% of the HeLa cells, and in presence of an unrelated control aptamer, Stx2-AF labeled 76% cells. Most of the Stx2-specific aptamers (mA_Apt1, mB_Apt23, mC_Apt41, mD_Apt46, pA_Apt1, and pCApt22) inhibited Stx2 binding weakly as approximately 50% of HeLa cells were labeled in the presence of these aptamers. Similar to the unrelated control aptamer, the remaining 2 Stx2-binding aptamers (pB_Apt14 and pD_Apt25) did not inhibit Stx2 binding. This experiment was repeated twice with similar results.

In absence of any aptamer, Stx2-AF (0.6 *μ*g/mL, 8 nM) labeled ~78% of the HeLa cells (Figure 3). Control aptamer did not block binding of Stx2 with HeLa cells as 76% cells were labeled by Stx2-AF. Most of the Stx2-specific aptamers (mA_Apt1, mB_Apt23, mC_Apt41, mD_Apt46, pA_Apt1, and pCApt22) inhibited Stx2 binding weakly as approximately 50% of HeLa cells were labeled in the presence of these aptamers (Figure 3). Similar to the unrelated control aptamer, the remaining 2 Stx2-binding aptamers (pB_Apt14 and pD_Apt25) did not inhibit Stx2 binding (Figure 3).

#### 3.2.2. Neutralization of Stx2-Mediated HeLa Cell Cytotoxicity by Aptamers

**Figure 4:**
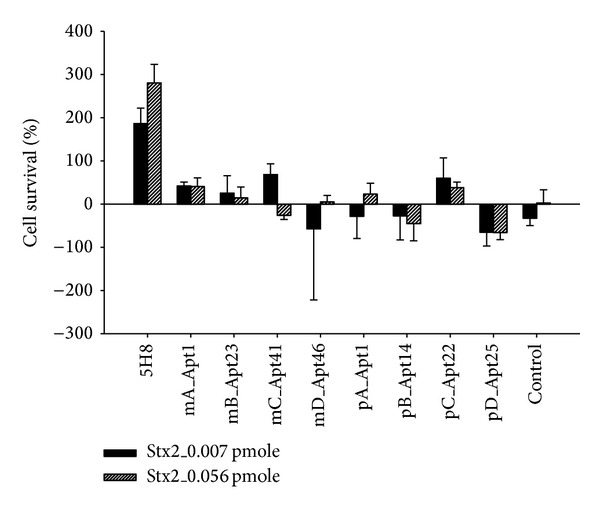
Neutralization of Stx2 activity by aptamers (1550 pmoles) in HeLa cell cytotoxicity Assay. Stx2-specific HuMAb 5H8 (6.66 pmole) strongly neutralized Stx2 activity at both toxin concentrations (0.007 pmole and 0.056 pmole). A limited neutralization of Stx2 activity was observed with aptamers mA_Apt1 (~40% cell survival), mB_Apt23 (~20% cell survival), and pC_Apt22 (≤60% cell survival). Aptamer mC_Apt41 neutralized 0.007 pmole of Stx2 (≤60% cell survival) but not 0.056 pmole of Stx2. None of the other aptamers neutralized the cytotoxic effects of Stx2. This experiment was repeated twice with similar results.

A limited neutralization of Stx2-mediated cytotoxicity and death of HeLa cells was observed with aptamers mA_Apt1 (~40% cell survival), mB_Apt23 (~20% cell survival), and pC_Apt22 (≤60% cell survival) (Figure 4). Aptamer mC_Apt41 neutralized 0.007 pmole of Stx2 (≤60% cell survival) but not 0.056 pmole of Stx2 (Figure 4). None of the other aptamers neutralized the cytotoxic effects of Stx2 (Figure 4). RNA aptamers did not degrade in the HeLa cell assay as RNA band of HeLa cell-incubated aptamer was of similar intensity in PAGE analysis as that of the unincubated aptamer (not shown).

## 4. Discussion

The goal of the present study was to identify aptamers that can bind and inactivate Stx1 and Stx2. The well known in vitro SELEX procedure [17, 18] was used to derive RNA aptamers that specifically bind to Stx1 and Stx2. Aptamer libraries without any modifications were used initially to demonstrate proof-in-principle that SELEX can be used to identify aptamers for Stx1 and Stx2. Generally, the aptamer pools with short randomized regions (15 nucleotides) are sufficient for a successful aptamer selection [30]. However, longer randomized regions give the pools a greater structural complexity [31, 32] and an increased possibility for selection of high-affinity aptamers [33]. Thus, we initially performed SELEX using a RNA pool containing 5 × 10^14^ unique sequences with a central stretch of 56 random nucleotides (N56) and identified aptamers that bound Stx2. However, despite using the same N56 pool and other pools with random nucleotide lengths (N35 and N101) and the same selection conditions as used for Stx2, we could not identify aptamers that bound Stx1. Although reasons for not identifying Stx1-binding aptamers are unclear, it can be assumed that Stx1 may not be a “good” target as evidenced by other studies that not every target is suitable for aptamer selection [32, 34].

Nucleic acid aptamers with binding affinities comparable to those of antibodies have been utilized for flexible applications ranging from diagnostic to therapeutic assay formats. To our knowledge this is the first study that attempted to discover aptamers against Stx2 and identified 6 unique unmodified RNA aptamer groups with Stx2-binding abilities. Although the Stx2-binding aptamers were discovered using N56 aptamer library, almost all aptamers had 39 random nucleotides except the group pD aptamers which had 56 random nucleotides. This is a common occurrence [35, 36] which might be because of the accumulation of either near-full length or nonfull length products in the pool during synthesis or primers recognizing the sequence of nucleotides similar to constant region in the randomized region [37].

All aptamers that bound Stx2 in the filter-binding assay also inhibited Stx2 binding partially with the HeLa cells except the aptamers of groups pB and pD which did not inhibit Stx2 binding. As antibodies that bind Stx2 B subunit but not A subunit are known to inhibit binding of the toxin with its cell surface [29], aptamers of groups mA, mB, mC, mD, pA, and pC that inhibited Stx2 binding may bind Stx2 B subunit. The Stx2-inhibiting aptamers seem to be of low affinity as very high concentrations (1550 pmole) of the aptamers relative to Stx2 concentrations (0.8 pmole) could not inhibit toxin binding completely. The affinity of aptamer was not increased as unmodified aptamers are nuclease sensitive which makes them unsuitable for therapy. These aptamers lost their ability to bind with Stx2 following modification (by incorporating Flourine at the 2′ position in pyrimidine bases) to gain nuclease resistant.

Very high concentrations of aptamers (1550 pmole) neutralized two different concentrations of Stx2 (0.007 pmole and 0.056 pmole) only marginally in HeLa cell assay. In contrast, the HuMAb 5H8 at 6.66 pmole (232 times lower than the aptamer concentrations) completely neutralized Stx2 at the two toxin concentrations. As very high concentrations of aptamers did not neutralize toxin's activity completely, dose response studies with aptamers were not performed. The insignificant neutralization of the toxin was not due to degradation of the aptamers as HeLa cell assay was performed in nuclease free conditions, and RNA aptamers did not degrade following incubation with HeLa cells.

Although we standardized SELEX to select Stx2-specific unmodified aptamers, our repeated attempts with modified libraries generated using 2′-Fluorine-CTP (2′-F-dCTP) and 2′-Fluorine-UTP (2′-F-dUTP) to identify nuclease resistant RNA aptamers against Stx1 or Stx2 were unsuccessful. As the 2′-fluoro modifications on the ribose ring influence the tertiary structure of an oligonucleotide, it may be expected that modified aptamers form completely unrelated structures compared to unmodified aptamers [23]. Moreover, we could not enrich the libraries with Stx2- or Stx1-binders as background binding was always as much as binding with Stx2 or Stx1 even after 12 rounds and, in a couple of instances, 20 rounds, of selection which might be because of increased hydrophobicity due to replacement of OH with fluorine in the RNA. This shows that SELEX technology is hard to adapt to select aptamers against some targets, such as Stx [34].

## 5. Conclusions

SELEX procedures, p-SELEX and m-SELEX, yielded unmodified aptamers that bound Stx2 but not Stx1. Each procedure generated 2 groups of Stx2-binding aptamer sequences that were unique to each SELEX procedure and another 2 groups of Stx2-binding aptamer sequences that were shared by the 2 SELEX procedures. These aptamers may be useful to develop Stx2-specific diagnostic assays in the future. Several attempts with modified RNA aptamer libraries to generate nuclease resistant aptamers against Shiga toxins were unsuccessful.

## Figures and Tables

**Table 1:** Concentrations of RNA aptamer pool and Stx1 or Stx2 for SELEX selection.

Selection round	Membrane-SELEX		Plate-SELEX	
	Input RNA (pmoles)	Input Stx1/Stx2 (pmoles)	Input RNA (pmoles)	Input Stx1/Stx2 (pmoles)
1	1600	400	400	28
2	800	400	200	28
3	200	200	200	28
4	200	200	200	28
5	200	50	200	28
6	200	50	200	28
7	200	25	200	28

**Table 2:** Sequences of RNA aptamers selected following membrane- and plate-SELEX.

Group	*n* ^ 1^	Sequence^2^
mA	43	ATTAGCTATCTTCCACGATTCGATCAGGCAGTACGTCGT^3,4^
mB	35	ACAGTTATCCGACTGCTATTCGATCAGGCAGTACGTAGC^3,5^
mC	6	CAGGCTGTTCTGACGCATAAGGAATGCGCTGTTGCAGAG
mD	4	TTGGTCCTGCTTTGGATAGTCGCGAAAGGGGTGCCACTG
m-Singles	8	Orphan sequences^6^
pA	29	ACAGTTATCCGACTGCTATTCGATCAGGCAGTACGTAGC^3,5^
pB	18	ACCGAGCGGTTTTACGTCTCAAGTAGTATCCCGTTTTGC
pC	7	ATTAGCTATCTTCCACGATTCGATCAGGCAGTACGTCGT^3,4^
pD	4	TTGCCATCCTGTACTATGCTCTATCGGGCGGTTTAGTGATCCTTCGTCCAACTATC
p-Singles	42	Orphan sequences^5^

^1^Number of isolates harboring the represented sequence in each group. ^2^Complete random sequences of the aptamer core regions (5′-3′) are shown. ^3^Underlined sequence of 19 bases is identical among groups mA, mB, pA and pC. ^4^Complete sequences of mA and pC groups are identical. ^5^Complete sequences of mB and pA groups are identical. ^6^Orphan sequences are the sequences that are seen only in one isolate.
